# NK cell phenotypic profile during active TB in people living with HIV-evolution during TB treatment and implications for bacterial clearance and disease severity

**DOI:** 10.1038/s41598-023-38766-7

**Published:** 2023-07-20

**Authors:** Thando Glory Maseko, Santhuri Rambaran, Slindile Ngubane, Lara Lewis, Sinaye Ngcapu, Razia Hassan-Moosa, Derseree Archary, Rubeshan Perumal, Nesri Padayatchi, Kogieleum Naidoo, Aida Sivro

**Affiliations:** 1 Centre for the AIDS Programme of Research in South Africa (CAPRISA), Durban, South Africa; 2 Department of Medical Microbiology, University of KwaZulu-Natal, Durban, South Africa; 3 South African Medical Research Council (SAMRC)-CAPRISA-TB-HIV Pathogenesis and Treatment Research Unit, University of KwaZulu-Natal Nelson R Mandela School of Medicine, Durban, South Africa; 4 JC Wilt Infectious Disease Research Centre, National Microbiology Laboratory, Public Health Agency of Canada, Winnipeg, MB Canada; 5 Department of Medical Microbiology and Infectious Diseases, University of Manitoba, Winnipeg, MB Canada

**Keywords:** Tuberculosis, HIV infections, Immunology, Innate immune cells

## Abstract

Natural killer (NK) cells, key effector cells of the innate immune system, play an important role in the clearance and control of *Mycobacterium tuberculosis* and HIV infections. Here, we utilized peripheral blood specimens from the Improving Retreatment Success CAPRISA 011 study to characterize NK cell phenotypes during active TB in individuals with or without HIV co-infection. We further assessed the effects of TB treatment on NK cell phenotype, and characterized the effects of NK cell phenotypes during active TB on mycobacterial clearance and TB disease severity measured by the presence of lung cavitation. TB/HIV co-infection led to the expansion of functionally impaired CD56^neg^ NK cell subset. TB treatment completion resulted in restoration of total NK cells, NK cell subset redistribution and downregulation of several NK cell activating and inhibitory receptors. Higher percentage of peripheral CD56^bright^ cells was associated with longer time to culture conversion, while higher expression of NKp46 on CD56^dim^ NK cells was associated with lower odds of lung cavitation in the overall cohort and the TB/HIV co-infected participants. Together these results provide a detailed description of peripheral NK cells in TB and TB/HIV co-infection and yield insights into their role in TB disease pathology.

## Introduction

Natural killer (NK) cells are lymphocytes of the innate immune system that have both cytotoxic and cytokine-producing effector functions and play a critical role in the recognition and destruction of infected and cancerous cells^1^. NK cells are classified into two functional subsets based on CD56 and CD16 surface marker expression. Cytokine-producing CD56^bright^ NK cells have a mainly immunomodulatory function and CD56^dim^ NK cells are primarily cytotoxic effector cells^2^. NK cells distinguish healthy cells from ‘stressed/infected/malignant’ cells based on the expression of surface activating and inhibitory receptors, which engage with target cells to regulate NK cell activity^3^. These responses play a key role in the pathogenesis of intracellular pathogens such as *Mycobacterium tuberculosis* (Mtb) and human immunodeficiency virus (HIV)^4,5^.

The importance and role of NK cell responses in Mtb infection and disease has been demonstrated by a number of in vitro, animal, clinical and epidemiological studies^6,7^. Early clearance of Mtb infection was proposed to be mediated by NK cells through recognition and lysis of Mtb and Mtb-infected cells^8–10^.NK cells are known to lyse Mtb-infected monocytes with NK cell activating receptors NKp46 and NKG2D playing a key role in this process^8,9,11^. Additionally, NK cells can interact and respond to Mtb through direct recognition of Mtb cell wall components by NKp44^12^ and TLR2 which results in Mtb killing^10^. Mtb infection is known to modify NK cell phenotype and function through downregulation of activating NK cell receptors (including NKp46) and expansion of anergic CD56^neg^ NK cell subsets^13^. A longitudinal study from South Africa has linked peripheral NK cell profiles and frequencies with TB disease progression, treatment response, and lung pathology^14^. Decreased NK cell frequency was associated with active TB disease progression, while increased NK cell levels and function was reported following successful TB treatment completion^14–16^. Changes in peripheral NK cell levels and activity are thought to reflect Mtb activity in the lung, with peripheral NK cell frequency during active TB being inversely correlated to the inflammatory burden in the lung^14^. Increased NK cell-mediated cytotoxicity was also associated with increased presence of lung cavitation, further implicating NK cells in lung immunopathology^17^.

In HIV infection, NK cells have been shown to play an important role in the early control of viral replication and disease progression. Increased proliferation of cytotoxic NK cells during the early stages of infection has been associated with long-term viral control in antiretroviral naïve individuals^18^. Inducible expression of natural cytotoxicity receptors NKp30 and NKp46 and increased Interferon-gamma (IFN-γ) production following NK cell activation correlates inversely with the size of the HIV-1 viral reservoir^19^. The activating killer cell immunoglobulin-like receptor (KIR) allele KIR3DS1, in combination with HLA-B alleles that encode molecules with isoleucine at position 80 (HLA-B Bw4-80Ile), was shown to be associated with delayed progression to AIDS^20^. HIV infection causes profound changes in the phenotypes and functions of NK cells and these changes are thought to hinder disease control^21^. This includes an HIV-mediated decrease in the expression of NKp44, NKp46, and NKp30 natural cytotoxicity receptors and the expansion of the anergic CD56^−^CD16^+^ NK cell subset^21^.

The convergence of the TB and HIV epidemics in sub-Saharan Africa has devastating consequences. TB is the leading cause of death in people living with HIV (PLHIV)^22,23^ and PLHIV are 18 times more likely to develop TB than people without HIV infection^24^. Despite the heightened need to elucidate the immunopathogenesis of TB/HIV, there remains limited data on NK cell phenotypes and immune responses in TB/HIV co-infection^25–27^. Here, we utilized specimens from the Improving Retreatment Success (IMPRESS, CAPRISA 011) study to characterize the effects of TB treatment on NK cell phenotype, and to characterize and assess the effects of NK cell phenotypes during active TB on mycobacterial clearance and TB disease severity in patients with or without HIV co-infection.

## Methods

### Study population

This sub-study included stored peripheral blood mononuclear cells (PBMCs) from 70 HIV-infected and uninfected adult participants from the CAPRISA 011 IMPRESS study at active TB (prior to treatment initiation) and at treatment completion time points based on sample availability. The CAPRISA 011 IMPRESS study was an open-label randomized control trial conducted at the CAPRISA eThekwini HIV-tuberculosis clinic in Durban, South Africa^28^. The IMPRESS trial compared the effectiveness of an interventional moxifloxacin-containing treatment to standard TB treatment for the improvement of culture conversion rates in smear-positive pulmonary TB^28^. TB treatment in the trial consisted of a two-month intensive treatment phase and four-month continuous phase. Recruited participants were ≥ 18 years with a history of anti-TB treatment and a current TB diagnosis as confirmed by either a positive culture of MTB in sputum or by GeneXpert MTB/RIF® technology (Cepheid, USA). Additionally, 13 PBMC samples from healthy controls from KwaZulu-Natal, South Africa were included in this study.

### Ethics statement

Ethics approval for the CAPRISA 011 Improving Retreatment Success (IMPRESS) Trial was obtained from the University of KwaZulu-Natal (UKZN) Biomedical Research Ethics Committee (BFC029/13; Clinical trial.gov; NCT02114684) and the South African Medicines Control Council (Ref:20130510). The protocol for this sub-study was approved by the UKZN Biomedical Research Ethics Committee (BREC/00002197/2020). Ethical approval for the use of PBMC samples from healthy controls from KwaZulu-Natal, South Africa was obtained from University of KwaZulu-Natal Biomedical Research Ethics Committee (BE432/12). All study participants provided written informed consent prior to enrolment and approval for the use of stored biological specimens for future research. All the experiments were performed in accordance with the Helsinki Declaration.

### Sample collection and processing

Peripheral blood was collected in acid citrate dextrose tubes and PBMCs were isolated by Ficoll-Hypaque density gradient centrifugation^29^. Isolated PBMCs were cryopreserved in fetal bovine serum (FBS) with 10% (v/v) DMSO and stored in liquid nitrogen until use.

### Flow cytometry experiments

Cryopreserved PBMC samples were thawed using a 37ºC water bath, washed, and resuspended in 5 ml R10 media (RPMI 1640 medium containing 2 mM L-glutamine and 25 mM HEPES buffer supplemented with 10% v/v FBS, 100 U/ml penicillin and 100 ug/ml streptomycin (Lonza). The resuspended cells were rested at 37ºC in 5% CO_2_ for 3 h. One million cells per panel were resuspended in 10% mouse IgG in PBS-2 (PBS supplemented with 2% FBS) and incubated for 15 min in the dark at 4ºC. The cells were pelleted by centrifugation, and surface stained per panel for 20 min at room temperature in the dark. Flow cytometry panels are described in Supplementary table 1. Panels included a LIVE/DEAD fixable dead cell stain (ThermoFisher Scientific, L34957) that was diluted with 50 µl of DMSO as per manufacturer’s instructions. The stock was diluted 1/40 and 3 µl of the dilution was used for final staining (per 50 µl reaction). Following staining, cells were washed twice using PBS-2 and resuspended in 1X CellFix™ (BD). Minimum of 200,000 events were acquired on a flow cytometer (BD LSRFortessa™ Cell analyzer, USA) using BD FACSDiva software v8.0.2. with the data analyzed using FlowJo software version 10.8.1.

### Flow cytometry gating strategy

The NK cell gating strategy used to identify total NK cells and NK cell subsets is presented in Supplementary Fig. 1. Total NK cells were identified as live CD3^−^CD14^−^CD19^−^ cells expressing CD56 and/or CD16. Total NK cells were further separated into the following subsets based on CD56 and CD16 expression: CD56^bright^ (CD56^++^CD16^+/−^), CD56^dim^ (CD56^+^CD16^++^), CD56^neg^ (CD56^−^CD16^+^) and CD56^dim^CD16^−^. We further characterized NK cell surface marker expression (Supplementary Fig. 2) on transcriptionally distinct CD56^bright^ and CD56^dim^ NK cell subsets^30^. Surface markers included activating and inhibitory NK cell surface receptors (NKG2D, NKG2C, NKG2A, NKp30, NKp44, NKp46, NKB1, and CD158)^30^.

### Statistical analysis

The statistical analyses were conducted using IBM SPSS Statistics v27 (Armonk, NY) and figures were compiled using GraphPad Prism v9.3.1. The distribution of the data was assessed using the D’Agostino-Pearson omnibus normality test. To investigate differences in the percentage (%) of total NK cells, % NK cell subsets, and differential NK cell marker expression across participant subgroups, one-way ANOVA with Tukey’s multiple comparisons test and Kruskal Wallis test with Dunns multiple comparisons test were used on normally distributed and non-normally distributed data, respectively. Depending on the distribution of the data, a paired t-test or Wilcoxon signed-rank test were used to assess the difference between the proportion of total NK cells, NK cell subsets, and surface marker expression during active TB (prior to treatment initiation) and following treatment completion. The relationship between NK cell frequencies and NK cell surface marker expression at active TB with days to culture conversion (defined as two consecutive negative TB sputum culture results) was modelled using Cox proportional hazards models. Those who died were excluded from the analysis while those that did not culture convert before leaving the study were censored on their study termination date. Logistic regression was used to determine the association between NK cell frequencies and NK cell surface marker expression during active TB with the presence of lung cavitation at baseline, an indicator of TB disease severity. The multivariable analyses adjusted for baseline clinical and demographic variables, including treatment randomization arm, age, gender, and HIV status. The sub-analysis of the TB/HIV co-infected group adjusted for treatment randomization arm, age, and gender.

## Results

### Cohort characteristics

The CAPRISA 011 IMPRESS cohort (n = 70) was comprised of 75.7% males with a median age of 35.5 years [interquartile range (IQR) 29–43)] and median BMI of 19.6 kg/m^2^ [interquartile range (IQR) 18.2–22.4)], (Table 1). The study included 44 (62.9%) HIV positive and 26 (37.1%) HIV negative participants with active TB. The HIV positive participants had a median CD4 count of 285 cells/mm^3^ (IQR 123–413) and 50% were on antiretroviral therapy (ART). Overall, 62.9% of the participants had lung cavitation in one or both lungs. The healthy donors consisted of 54% females with a median age of 35 years (IQR 32–39) and median BMI of 23.29 kg/m2 (IQR 19.73–31.30), Supplementary table 2.

**Table 1:** Demographic and clinical characteristics of CAPRISA 011 IMPRESS participants.

Variables	Total cohort n = 70	HIV positive n = 44	HIV negative n = 26
Randomization arm n (%)			
HRZE—Control	29 (41.4)	17 (38.6)	12 (46.2)
HRZM—Active	41 (58.6)	27 (61.4)	14 (53.8)
Gender, n (%)			
Male	53 (75.7)	31 (70.5)	22 (84.6)
Age (y), median (IQR)	35.5 (29.0–43.0)	37.0 (31.0–41.0)	33.0 (24.8–50.5)
Body mass index (kg/m^2^), median (IQR)	19.6 (18.2–22.4)	19.7 (18.1–22.5)	19.3 (18.4–22.2)
HIV status, n (%)			
Positive	44 (62.9)	44 (100.0)	–
Negative	26 (37.1)	–	26 (100.0)
HIV Viral load (copies/ml), median (IQR)	–	45,165.0 (2857.3–558,065.0)	–
CD4 cell count (cells/mm^3^), median (IQR)	–	285 (123–413)	–
ARV status^a^, n (%)			
Yes	–	22 (50.0)	
No	–	20 (45.5)	
Lung Cavities^b^, n (%)			
None	18 (25.7)	13 (29.5)	5 (19.2)
One Lung	24 (34.3)	17 (38.6)	7 (26.9)
Both Lungs	20 (28.6)	11 (25.0)	9 (34.6)
Alcohol Use in the past 3 months n (%)			
Yes	17 (24.3)	14 (31.8)	3 (11.5)
Smoking in past 3 months n (%)			
Yes	25 (35.7)	15 (34.1)	10 (38.5)

^#^Values for clinical and demographic variables reported at baseline except days to first negative culture.

^a^Two participants missing ARV status.

^b^Eight participants with missing lung cavitation status.

### Effect of active TB and TB/HIV co-infection on NK cell percentage and phenotype

**Figure 1:**
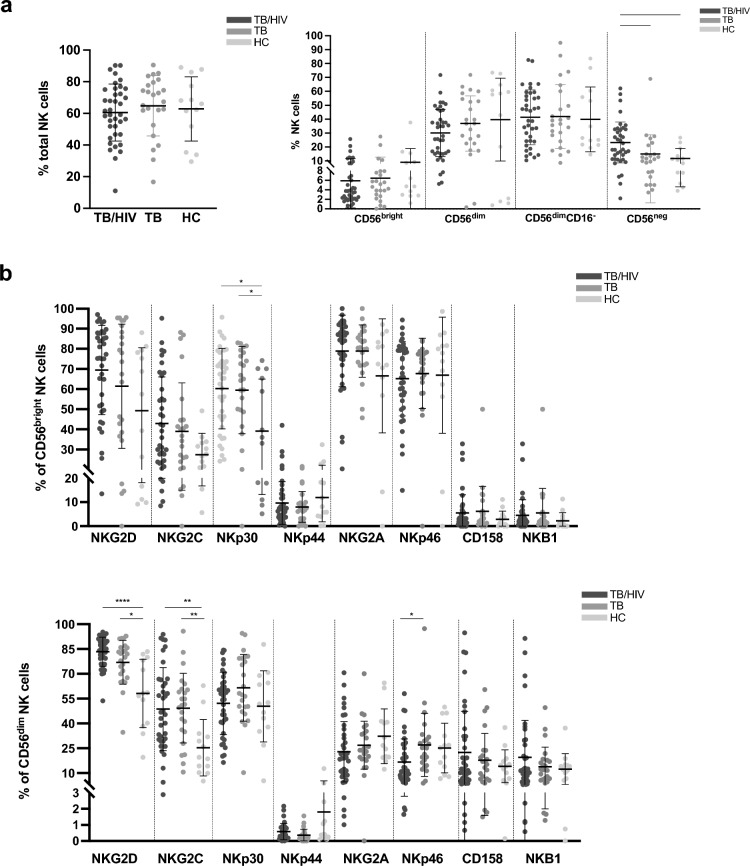
Differences in NK cell percentage and phenotype between TB/HIV co-infected and TB infected participants and healthy controls. (**a**) Differences in the % of total NK cells and NK cell subsets across participant groups (TB/HIV, TB, healthy controls). (**b**) Percentage of NK cell activating and inhibitory receptors on CD56^bright^ and CD56^dim^ NK cells across participant groups (TB/HIV, TB, healthy controls). *p < 0.05; **p < 0.01, ***p < 0.001 and ****p < 0.0001.

We compared the percentage of NK cell populations and NK cell surface receptor expression across the following participant groups: CAPRISA 011 TB/HIV co-infected participants (TB/HIV), CAPRISA 011 HIV-negative participants with active TB (TB), and healthy controls (Fig. 1). The TB/HIV group had a significantly higher % of CD56^neg^ NK cells compared to the TB group (*p* = 0.0176) and healthy controls (*p* = 0.0148) (Fig. 1a).

The expression of cytotoxicity receptor NKp30 on CD56^bright^ NK cells was significantly higher in the TB/HIV (*p* = 0.0348) and TB (*p* = 0.0445) groups in comparison to the healthy controls (Fig. 1b). NKG2D expression on CD56^dim^ NK cells was significantly higher in TB/HIV (*p* = 0.0001) and TB group (*p* = 0.0308) in comparison to healthy controls. Similarly, NKG2C expression on CD56^dim^ NK cells was significantly higher in TB/HIV (*p* = 0.0055) and TB group (*p* = 0.0084) in comparison to healthy controls. A decrease in NKp46 on CD56^dim^ NK cells in TB/HIV group compared to TB group was observed (*p* = 0.0171) (Fig. 1b).

### Changes in NK cell percentage and phenotype following TB treatment completion

**Figure 2:**
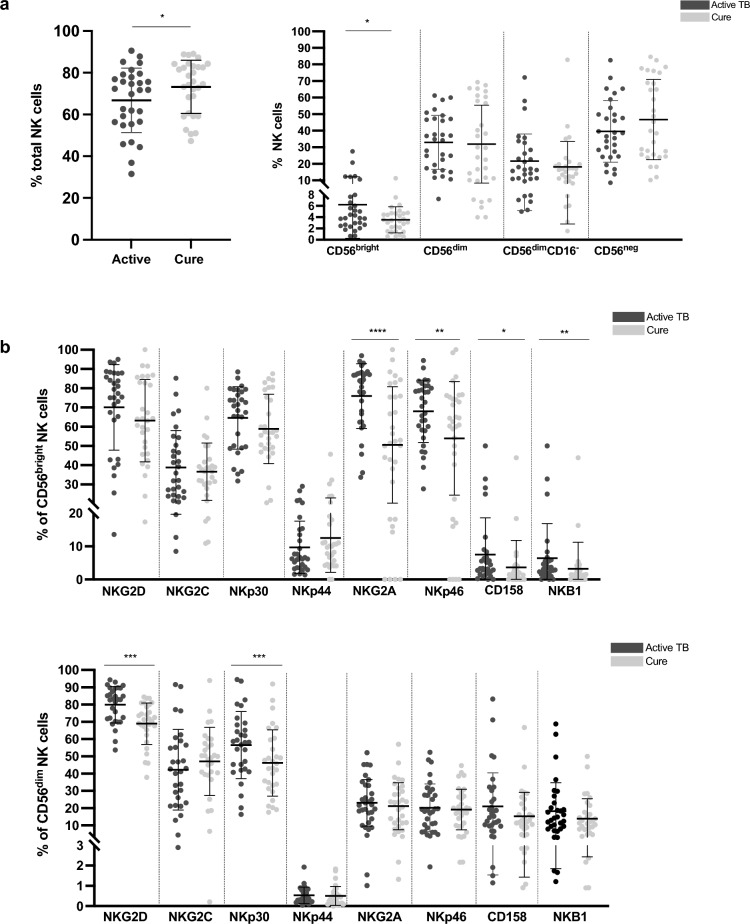
Effect of TB treatment completion on NK cell percentage and phenotypic marker expression in the CAPRISA 011 (n = 34). (a) Effect of TB treatment completion on the percentage of total NK cells and NK cell subsets. (b) Differences in activating and inhibitory receptor expression on CD56^bright^ and CD56^dim^ NK cells between active TB and post treatment completion. *p < 0.05; **p < 0.01, ***p < 0.001 and ****p < 0.0001.

The differences in the percentage of NK cells and NK cell surface receptor expression between active TB and post-treatment completion were examined in 34 paired samples (Fig. 2). Following treatment completion, we observed a significant increase in the total NK cell population (*p* = 0.024) (Fig. 2a). With respect to NK cell subsets, we observed a decrease in CD56^bright^ NK cells following treatment completion (*p* = 0.032), in the total cohort.

Several NK cell surface markers were downregulated on CD56^bright^ NK cells following treatment completion, including NKG2A (*p* = 0.0001), NKp46 (*p* = 0.003), CD158 (*p* = 0.026), and NKB1 (*p* = 0.006). On CD56^dim^ NK cells, we observed a significant downregulation of NKG2D (*p* = 0.0001) and NKp30 (*p* = 0.001) following TB treatment completion in the total cohort (Fig. 2b).

Similar differences and trends were observed in HIV positive sub-group (Supplementary Fig. 3). In addition, NKp44 was significantly upregulated in HIV positive participants on CD56^bright^ NK cells following treatment completion (*p* = 0.049). On CD56^dim^ NK cells, there was an additional significant decrease in CD158 (*p* = 0.003) and an increase in NKG2C (*p* = 0.013) expression following treatment completion.

### Association between NK cell percentage and phenotype during active TB and time to culture conversion

We utilized a cox regression model to assess the association between NK cell percentage and phenotype at active TB on time to negative culture conversion, (Table 2, Supplementary table 4, Supplementary Fig. 4). Higher % of CD56^bright^ NK cells was associated with longer time to culture conversion [adjusted hazards ratio (aHR) 0.893, 95% CI: 0.825–0.966, *p* = 0.005] while the higher % of CD56^neg^ NK cells was associated with shorter time to culture conversion (aHR 1.029, 95% CI: 1.006–1.052, *p* = 0.013) in the multivariable model controlling for the treatment randomization arm, age, gender, and HIV status. Increased expression of NKG2D on CD56^dim^ NK cells was associated with longer time to culture conversion (aHR 0.961, 95% CI: 0.932–0.990, *p* = 0.0085) (Table 2, Supplementary table 4).

**Table 2:** Significant association between NK cells and NK phenotypic markers with time to culture conversion (n = 63).

Cohort	Subset	Cell marker	Bivariable			Multivariable		
			HR	CI	*p*-value	aHR	CI	*p*-value
Total cohort	% of CD56^bright^ NK cells		0.939	0.877–1.007	0.077	0.893	0.825–0.966	**0.005**
	% CD56^neg^ NK cells		1.022	0.999–1.045	0.063	1.029	1.006–1.052	**0.013**
	% of CD56^dim^ NK cells	NKG2D	0.977	0.953–1.001	0.064	0.961	0.932–0.990	**0.009**
TB/HIV co-infected	% of CD56^bright^ NK cells		0.951	0.882–1.026	0.196	0.858	0.768–0.959	**0.007**
	% of CD56^bright^ NK cells	NKp46	0.997	0.977–1.018	0.794	0.975	0.952–0.999	**0.045**

Significant values are in [bold].

We observed a similar trend in the sub-analysis of HIV co-infected participants (n = 39) with the higher % of CD56^bright^ NK cells being associated with longer time to culture conversion (aHR 0.858, 95% CI: 0.768–0.959, *p* = 0.007). Increased expression of NKp46 on CD56^bright^ NK cells was associated with longer time to culture conversion (aHR 0.975, 95% CI: 0.952–0.999, *p* = 0.045), (Supplementary table 5).

### Association between NK cell percentage and phenotype during active TB and cavitary disease

Binary logistic regression models were used to assess the association of NK cell percentage and phenotype on TB disease severity measured by presence of lung cavitation (Table 3, Supplementary table 6). Higher expression of NKp30 on CD56^bright^ NK cells increased the odds of lung cavitation in the total cohort [adjusted odds ratio (aOR) 1.036, 95% CI: 1.002–1.071, *p* = 0.039)]. Higher expression of NKp46 on CD56^dim^ NK cells lowered the odds of lung cavitation in the total cohort (aOR 0.958, 95% CI: 0.921–0.997, *p* = 0.035) and among the TB/HIV co-infected participants (aOR 0.937, 95% CI: 0.883–0.994, *p* = 0.032), (Table 3, Supplementary tables 6 and 7).

**Table 3:** Significant associations between frequencies of NK cells and NK phenotypic markers with disease severity measured by lung cavitation (n = 55).

Cohort	Subset	Cell marker	Bivariable			Multivariable		
			OR	CI	*p*-value	aOR	CI	*p*-value
Total cohort	% of CD56^bright^ NK cells	NKp30	1.028	0.998–1.058	0.068	1.036	1.002–1.071	**0.039**
	% of CD56^dim^ NK cells	NKp46	0.971	0.937–1.006	0.103	0.958	0.921–0.997	**0.035**
TB/HIV co-infected	% of CD56^dim^ NK cells	NKp46	0.943	0.892–0.998	**0.044**	0.937	0.883–0.994	**0.032**

Significant values are in [bold].

## Discussion

Both Mtb and HIV cause alterations in the NK cell repertoire with consequences for pathogen control and disease outcome. In this study, we assessed the effects of TB treatment on NK cell percentage and phenotype, and the characteristics and effects of NK cell phenotypes during active TB disease on bacterial clearance and disease severity in patients with or without HIV co-infection.

We observed an expansion of the CD56^neg^ NK cells in the TB/HIV group compared to TB group and healthy controls. This is consistent with previous reports showing an increased proportion of CD56^neg^ NK cell subset during both HIV and TB infections^31–34^. Expansion of the CD56^neg^ NK cell subset during HIV infection is associated with rapid and early progression to AIDS^35^. This NK cell subset was shown to have diminished cytolytic and cytokine-producing capacity, a likely sequela of NK cell exhaustion due to chronic inflammation resulting from prolonged viral and bacterial infections^31,36^.

We observed an increase in activating receptor NKp30 expression on immunomodulatory, weakly cytotoxic CD56^bright^ NK cell subsets in the TB and TB/HIV groups compared to healthy controls, potentially increasing the recognition and response against Mtb and HIV-infected cells. The CD56^dim^ NK cell subset had increased expression of activating receptors NKG2D and NKG2C in TB and TB/HIV group compared to healthy controls. An increase in NKG2D NK cell expression was reported following exposure to Mtb-infected monocytes^9^ pinpointing NKG2D as one of the principal receptors involved in the lysis of Mtb-infected mononuclear phagocytes. Expansion of NKG2C^+^ NK cells was previously reported in response to human cytomegalovirus (CMV) and HIV/ Simian immunodeficiency virus (SIV) infections^37–39^ and is thought to play an important role in viral control and slower disease progression ^39^. We additionally observed a decrease in NK cell activating receptor NKp46 on CD56^dim^ NK cell subsets in the TB/HIV group compared to the TB group. Expression of natural cytotoxicity receptors including NKp46 is known to be decreased among viremic HIV positive individuals with a concomitant decrease in NK cytolytic activity^40^. As NKp46 is involved in the lysis of Mtb-infected monocytes^8^, this HIV-mediated decrease in NKp46 could further impair NK cell-mediated Mtb control in TB/HIV co-infected patients.

Following TB treatment, we observed an increase in the total NK cell proportion and a decrease in CD56^bright^ NK cells. Decrease in total NK cells during active TB and their restoration following treatment have been reported previously^14,41^. Changes in peripheral NK cell proportions are thought to reflect bacterial burden and Mtb activity in the lungs, with an increase in NK cells being associated with successful treatment outcomes and a decrease in NK cells being associated with disease progression^14^. A decrease in CD56^bright^ NK cells during active TB has also been reported^13^, and this could potentially be due to a redistribution of NK cells by trafficking to the site of infection^14^ with an increase in CD56^bright^ NK cells observed in the pleural fluid of TB patients^42^.

Successful treatment completion resulted in downregulation of several NK cell activating receptors including NKp46 levels on CD56^bright^ NK cells and NKG2D and NKp30 levels on CD56^dim^ NK cells. We additionally observed a decrease in inhibitory receptors NKG2A, CD158 (KIR), and NKB1 on CD56^bright^ NK cells following treatment completion. In addition to the above, HIV co-infected participants had a significant increase in NKp44 levels on CD56^bright^ NK cells and an increase in NKG2C levels on CD56^dim^ NK cells following TB treatment completion. The observed decrease in the expression of NK cell activating receptors following treatment completion likely reflects the accompanying decrease in Mtb burden, with both NKG2D and NKp46 shown to play a key role in NK cell recognition and clearance of Mtb infected cells^8,9^.

Data on the role of inhibitory NK cell receptors in Mtb infection is limited. Inhibitory receptors like NKG2A function as immune checkpoints to prevent overactivation of the host immune system^43,44^. Viral infections, including HIV, are known to upregulate NKG2A expression on peripheral NK cells causing immunosuppression and reduction in NK cell cytotoxic activity^45–47^. Decreased NKG2A expression and increased KIR expression is also associated with NK cell maturation and differentiation^48,49^. Therefore, the observed decrease in inhibitory NK cell receptors following TB treatment completion likely corresponds to the decrease in Mtb burden in the lungs and reduction in NK cell activity.

Interestingly, in TB/HIV co-infected participants, a significant increase in NKp44 levels on CD56^bright^ NK cells and an increase in NKG2C levels on CD56^dim^ NK cells following TB treatment completion was found. Both NKp44 and NKG2C expression is associated with HIV and Mtb control. However, NKp44-expressing NK cells were also implicated in HIV disease progression, through lysis of NKp44 ligand expressing CD4^+^ T cells, a ligand that is specifically induced on CD4^+^ T cells from HIV-infected patients^50^. How TB treatment completion leads to increased expression of these activating NK receptors and their implications in HIV and TB pathogenesis remains to be determined.

An association between higher percentage of systemic anergic CD56^neg^ NK cells during active TB and shorter time to culture conversion was observed. Additionally, higher percentage of peripheral CD56^bright^ NK cells and higher expression of activating receptor NKG2D on CD56^dim^ NK cells were associated with longer time to culture conversion. One potential explanation for these associations could be the inverse relationship between percentage of these cells in the blood and in the lungs as they may be redistributed to the tissues of higher antigen concentration. This is supported by previous study showing an inverse correlation between peripheral NK cell frequencies and inflammatory burden in the lung^14^. Similarly, severe COVID-19 disease was associated with reduced frequencies of peripheral NK cells^51–53^ and increased frequency of NK cells in the lungs^54,55^. However, better understanding of composition and function of NK cells in lung tissues and how these relate to peripheral NK cells and TB pathology is needed.

We further observed an association between activating receptors NKp30 and NKp46 and the presence of lung cavitation. Higher expression of NKp30 on systemic CD56^bright^ NK cells during active TB was associated with increased odds of lung cavitation while the higher expression of NKp46 on systemic CD56^dim^ NK cells was associated with lower odds of lung cavitation. NK cells can aid in pathogen clearance however, they can also be rather detrimental and contribute to lung immunopathology. NK cell related inflammation and injury to the pulmonary structures was described in lung infection mouse model^56^, and increased NK cell activity in the blood has been previously associated with cavitary disease in humans^57^.

Our study has several limitations including the use of peripheral blood specimens to look at changes in lung immunopathology. Furthermore, our panels did not include an exhaustive list of activating and inhibitory receptors and due to limitations in cell numbers, we were unable to assess NK cell responsiveness. Despite these limitations, our data still shows distinct changes in systemic NK cell populations with respect to active disease, treatment completion, and disease severity in TB and TB-HIV co-infected individuals.

## Supplementary Information

Supplementary Information.

## Data Availability

The datasets used and/or analysed during the current study are available from the corresponding author on reasonable request.
