# Supplementary material for: NK cell phenotypic profile during active TB in people living with HIV-evolution during TB treatment and implications for bacterial clearance and disease severity
Source: Sci Rep. 2023 Jul 20;13:11726. doi: 10.1038/s41598-023-38766-7 (PMC10359304; doi:10.1038/s41598-023-38766-7)
Supplement: Supplementary file 1 — Supplementary Information. [file 41598_2023_38766_MOESM1_ESM.docx]

**Supplementary material**

**NK cell phenotypic profile during active TB in people living with HIV– evolution during TB treatment and implications for bacterial clearance and disease severity**

Thando Glory Maseko^1,3^, Santhuri Rambaran^1^, Slindile Ngubane^1^, Lara Lewis^1^, Sinaye Ngcapu^1,2^, Razia Hassan-Moosa^1,3^, Derseree Archary^1,2^, Rubeshan Perumal^1,3^, Nesri Padayatchi^1,3^, Kogieleum Naidoo^1,3^, Aida Sivro^1,2,3,4*^

^1^Centre for the AIDS Programme of Research in South Africa (CAPRISA), Durban, South Africa

^2^Department of Medical Microbiology, University of KwaZulu-Natal, Durban, South Africa

^3^South African Medical Research Council (SAMRC)-CAPRISA-TB-HIV Pathogenesis and Treatment Research Unit, University of KwaZulu-Natal Nelson R Mandela School of Medicine, Durban, South Africa

^4^JC Wilt Infectious Disease Research Centre, National Microbiology Laboratory, Public Health Agency of Canada, Winnipeg, MB, Canada

^5^Department of Medical Microbiology and Infectious Diseases, University of Manitoba, Winnipeg, MB, Canada

**Supplementary table 1**. Antibody panels for NK cell phenotypic characterization

| Panels | Antigen | Fluorochrome | Clone | Manufacturer | Catalogue No. |
| --- | --- | --- | --- | --- | --- |
| Panel 1 | CD3 | AFluor700 | SK | Biolegend | 344822 |
|  | CD19 | AFluor700 | HIB19 | BD Biosciences | 557921 |
|  | CD14 | AFluor700 | MSE2 | BD Biosciences | 557923 |
|  | CD16 | BV785 | 3G8 | Biolegend | 302046 |
|  | CD56 | PECy7 | B159 | BD Biosciences | 567747 |
|  | NKG2D | PerCPCy5.5 | 1D11 | BD Biosciences | 562364 |
|  | NKG2C | PE | 134591 | R&D Systems | FAB138P |
|  | NKp30 | BV711 | P30–15 | BD Biosciences | 536383 |
|  | NKp44 | APC | P44–8 | BD Biosciences | 558654 |
|  | Live/Dead | Amcyan | – | Thermofisher | L34957 |
| Panel 2 | CD3 | AFluor700 | SK | Biolegend | 344822 |
|  | CD19 | AFluor700 | HIB19 | BD Biosciences | 557921 |
|  | CD14 | AFluor700 | MSE2 | BD Biosciences | 557923 |
|  | CD16 | V450 | 3G8 | BD Biosciences | 560474 |
|  | CD56 | PECy7 | B159 | BD Biosciences | 567747 |
|  | CD335 (NKp46) | BV786 | PE2 | BD Biosciences | 563329 |
|  | CD159a (NKG2A) | APC | Z199 | Beckman Coulter | A60797 |
|  | CD158e | PE | Z27.3.7 | Beckman Coulter | IM3292 |
|  | NKB1 | FITC | Dx9 | BD Biosciences | 555966 |
|  | Live/Dead | Amcyan | – | Thermofisher | L34957 |


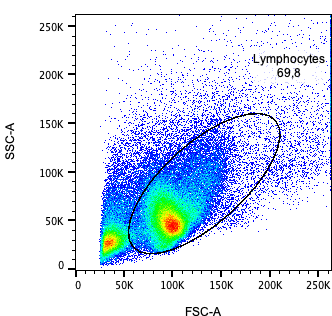

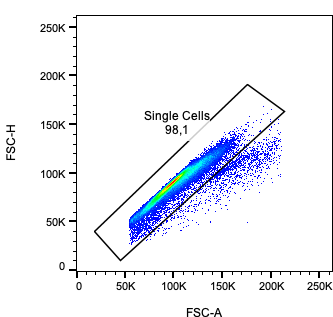

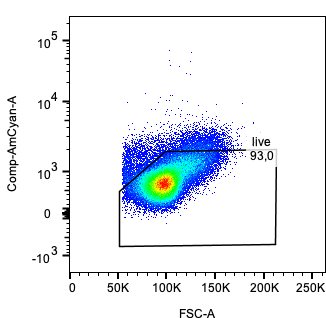

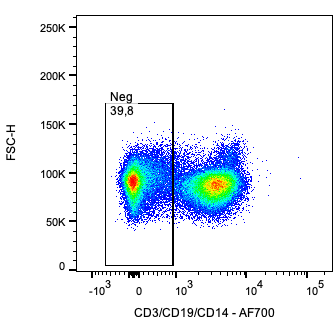

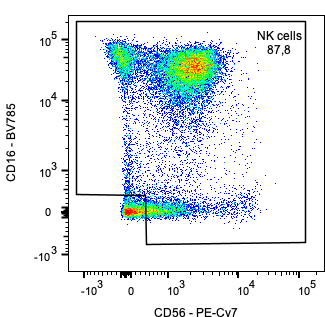

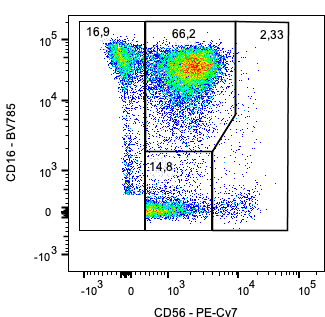


**Supplementary figure 1. Representative flow cytometry plot illustrating parent gating.** Lymphocytes were identified by plotting side scatter area (SCA) and forward scatter area (FCA). Single cells were identified by plotting forward scatter height (FCH) and area (FCA). Live cells were then identified by low expression of Aqua Dead cell stain. We then excluded cells with CD3, CD19 and CD14 surface markers by gating on the negative population. We identified the total NK cell population by gating on the CD56 and CD16 positive cells. These were further separated by gating on CD56^bright^, CD56^dim^, CD56^neg^ and the CD56^dim^CD16^–^ subsets.


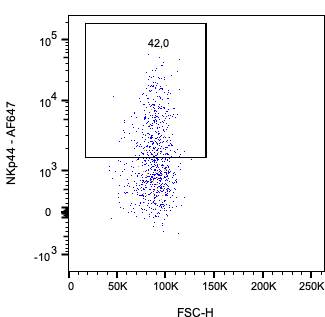

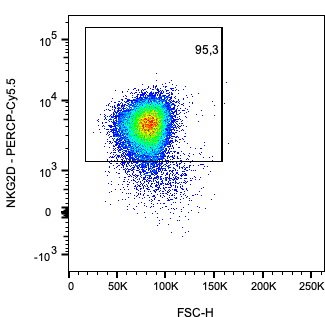

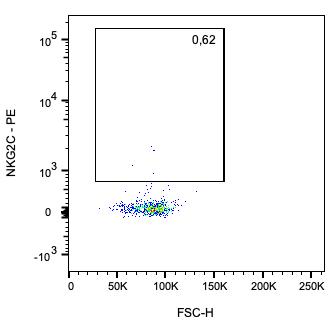

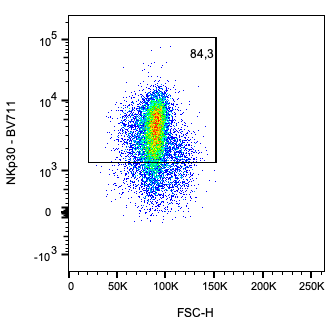

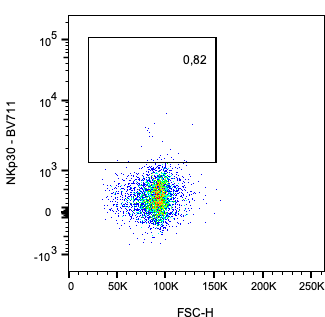

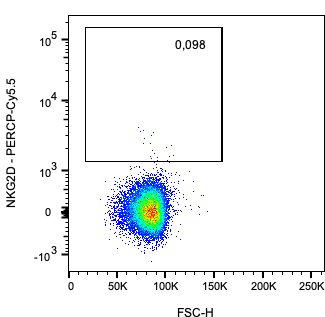

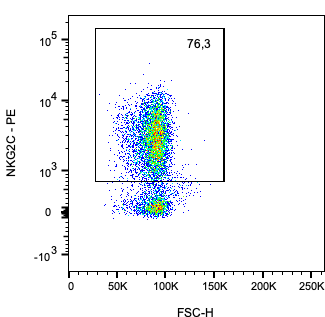

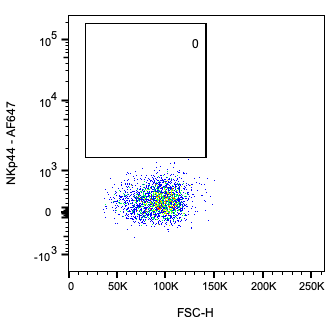

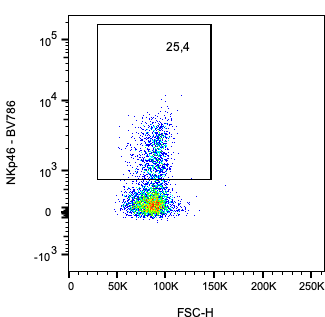

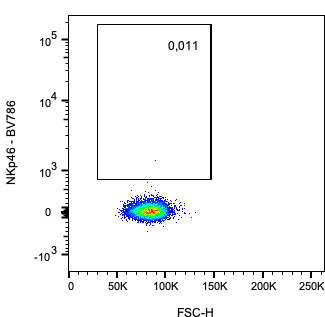

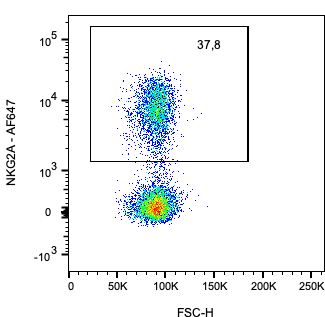

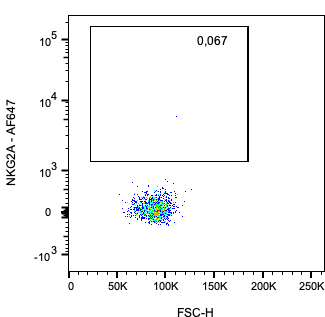

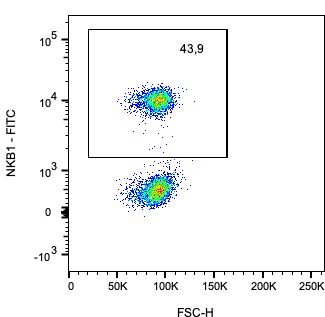

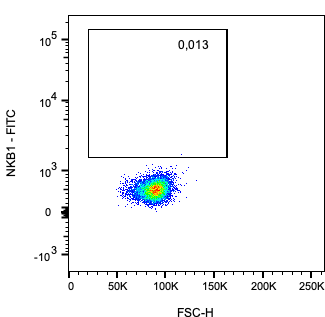

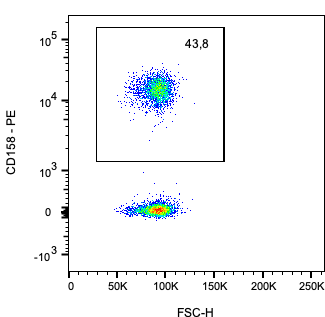

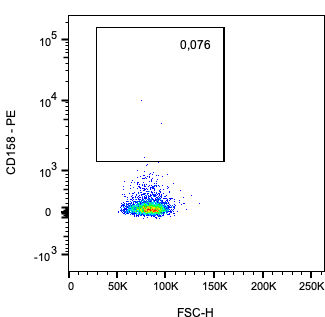


**a**

**b**

**Supplementary figure 2. Representative flow cytometry plots showing expression of NKG2D, NKG2C, NKp30, NKp44, NKp46, NKG2A, NKB1 and CD158 on peripheral blood mononuclear cells.** (a) Representative NK cell phenotypic marker expression from TB/HIV co–infected participant (b) Flourescence Minus One (FMO) controls.

**Supplementary table 2.** Demographic and clinical characteristics of healthy controls.

| **Variables** | **Healthy control**  **n = 13** |
| --- | --- |
| **Gender, n (%)** |  |
| Male | 7 (46) |
| Female | 6 (54) |
| **Age (y), median (IQR)** | 35 (32 – 39) |
| **Body mass index (kg/m^2^), median (IQR)** | 23.29 (19.73 – 31.30) |
| **HIV status, n (%)** |  |
| Positive |  |
| Negative | 13 |

**Supplemental table 3.** NK cell population frequencies among participant groups

|  | **TB/HIV** | **TB** | **HC** | **p-value** |
| --- | --- | --- | --- | --- |
|  | Median (IQR) | Median (IQR) | Median (IQR) |  |
| **% Total NK cells** | 60.35  (46.70 - 75.15) | 69.55  (54.85 - 79.05) | 66.00  (43.60 - 81.65) | 0.6841 |
| **% NK cells** |  |  |  |  |
| CD56^bright^ | 3.730  (2.133 - 8.225) | 4.895  (2.750 - 7.855) | 4.720  (2.875 - 12.37) | 0.4026 |
| CD56^dim^ | 26.80  (15.15 - 46.15) | 36.85  ( 22.50 - 54.10) | 57.70  (1.865 - 62.70) | 0.1926 |
| CD56^+^CD16^-^ | 42.45  (24.13 – 57.18) | 37.85  (25.18 – 57.48) | 26.50  (21.55 – 58.15) | 0.9604 |
| CD56^neg^ | 18.10  (11.20 - 32.70) | 11.40  (6.960 – 19.63) | 10.70  (6.450 – 16.20) | **0.0028** |
| **NK cell markers** | | | | |
| **CD56^bright^** |  |  |  |  |
| NKG2C | 37.55  (23.75 - 61.60) | 35.55  (21.65 - 44.75) | 27.70  (21.30 - 33.60) | 0.0927 |
| NKG2D | 75.30  (51.08 - 87.75) | 70.50  (37.08 - 90.70) | 57.50  (13.90 - 78.75) | 0.1092 |
| NKp30 | 65.35  (43.88 - 74.90) | 64.70  (48.93 – 77.68) | 36.00  (14.45 – 67.75) | **0.0264** |
| Nkp44 | 7.190  (3.683 - 13.05) | 7.095  (2.960 - 9.895) | 8.160  (4.150 - 20.50 | 0.6916 |
| NKG2A | 85.60  (74.60 - 91.00) | 80.50  (72.15 - 88.18) | 72.50  (61.35 - 85.30) | 0.1619 |
| NKp46 | 65.90  (54.20 - 80.00) | 73.00  (63.85 - 77.78) | 77.90  (59.30 - 84.25) | 0.5296 |
| CD158 | 2.905  (1.510 - 5.958) | 2.805  (1.043 - 6.200) | 1.130  (0.3100 - 4.280) | 0.3698 |
| NKB1 | 2.730  (1.360 - 5.000) | 2.175  (1.085 - 5.100) | 0.7200  (0.000 - 3.630) | 0.1430 |
| **CD56^dim^** |  |  |  |  |
| NKG2C | 46.10  (29.18 - 66.50) | 51.20  (35.28 - 60.68) | 19.50  (12.25 - 32.90) | **0.0045** |
| NKG2D | 84.85  (78.45 - 89.73) | 81.00  (68.18 - 85.88) | 58.30  (39.25 - 79.10) | **0.0001** |
| NKp30 | 55.60  (38.80 -64.50) | 57.75  (47.83 - 79.40) | 51.80  (36.50 - 66.70) | 0.1326 |
| NKp44 | 0.4400  (0.1800 - 0.8450) | 0.2800  (0.1550 -0.3950) | 0.3900  (0.2400 - 1.835) | 0.1540 |
| NKG2A | 19.00  (8.770 - 33.10) | 23.80  (18.53 - 35.00) | 26.70  (19.55 - 41.00) | 0.0958 |
| NKp46 | 11.10  (6.230 - 25.00) | 25.20  (15.05 - 31.43) | 25.30  (11.33 - 39.30) | **0.0106** |
| CD158 | 11.60  (5.860 - 32.20) | 14.30  (5.773 - 22.90) | 14.20  (6.135 - 17.95) | 0.9525 |
| NKB1 | 10.80  (4.980 - 27.00) | 11.80  (5.750 - 17.53) | 12.20  (6.110 - 14.80) | 0.9396 |

**Supplementary figure 3. Effect of treatment completion on NK cell percentage and phenotypic marker expression in TB/HIV co–infected participants (n = 26).** (a) Effect of treatment on the percentage of total NK cells and NK cell subsets. (b) Differences in activating and inhibitory receptor expression on CD56^bright^ and CD56^dim^ NK cells between active TB and post treatment completion. *p < 0.05; ** p < 0.01, *** p < 0.001 and ****p < 0.0001

**Supplementary figure 4.** Significant associations between peripheral NK cells and NK phenotypic markers and time to culture conversion. A) total cohort B) TB/HIV co-infected study participants.

**Supplementary table 4.** Association between NK cell phenotypes and time to culture conversion among the total cohort (n=63).

|  |  | **Bivariable** | |  |  | **Multivariable** | |  |
| --- | --- | --- | --- | --- | --- | --- | --- | --- |
|  | **Cell marker** | **HR** | **CI** | **p–value** |  | **aHR** | **CI** | **p–value** |
| % Total NK cells |  | 0.979 | 0.961 – 0.998 | **0.027** |  | 0.987 | 0.968 – 1.006 | 0.171 |
|  |  |  |  |  |  |  |  |  |
| % CD56^bright^ |  | 0.939 | 0.877 – 1.007 | 0.077 |  | 0.893 | 0.825 – 0.966 | **0.005** |
| % CD56^dim^ |  | 0.988 | 0.972 – 1.004 | 0.141 |  | 0.983 | 0.966 – 1.001 | 0.063 |
| % CD56^dim^CD16^–^ |  | 1.006 | 0.991 – 1.021 | 0.443 |  | 1.008 | 0.992 – 1.025 | 0.319 |
| % CD56^neg^ |  | 1.022 | 0.999 – 1.045 | 0.063 |  | 1.029 | 1.006 – 1.052 | **0.013** |
|  |  |  |  |  |  |  |  |  |
| % CD56^bright^ | NKG2C | 0.988 | 0.975 – 1.002 | 0.090 |  | 0.992 | 0.978 – 1.005 | 0.232 |
|  | NKG2D | 1.002 | 0.991 – 1.013 | 0.690 |  | 1.004 | 0.993 – 1.015 | 0.502 |
|  | NKp30 | 1.003 | 0.988 – 1.018 | 0.676 |  | 1.002 | 0.987 – 1.018 | 0.763 |
|  | NKp44 | 1.021 | 0.983 – 1.061 | 0.273 |  | 1.014 | 0.975 – 1.055 | 0.493 |
|  | NKB1 | 0.987 | 0.956 – 1.018 | 0.409 |  | 1.004 | 0.969 – 1.041 | 0.818 |
|  | NKG2A | 1.006 | 0.987 – 1.025 | 0.528 |  | 0.998 | 0.979 – 1.017 | 0.802 |
|  | NKp46 | 0.995 | 0.978 – 1.012 | 0.530 |  | 0.986 | 0.97 – 1.003 | 0.099 |
|  | CD158 | 0.994 | 0.966 – 1.023 | 0.692 |  | 1.015 | 0.982 – 1.049 | 0.380 |
|  |  |  |  |  |  |  |  |  |
| % CD56^dim^ | NKG2C | 1.004 | 0.992 – 1.017 | 0.467 |  | 1.007 | 0.994 – 1.019 | 0.304 |
|  | NKG2D | 0.977 | 0.953 – 1.001 | 0.064 |  | 0.961 | 0.932 – 0.990 | **0.009** |
|  | NKp30 | 0.993 | 0.979 – 1.008 | 0.376 |  | 0.994 | 0.978 – 1.01 | 0.462 |
|  | NKp44 | 1.024 | 0.513 – 2.046 | 0.946 |  | 0.769 | 0.37 – 1.598 | 0.481 |
|  | NKB1 | 0.997 | 0.983 – 1.011 | 0.691 |  | 0.999 | 0.985 – 1.015 | 0.943 |
|  | NKG2A | 0.994 | 0.977 – 1.011 | 0.507 |  | 0.990 | 0.972 – 1.008 | 0.283 |
|  | NKp46 | 1.004 | 0.986 – 1.021 | 0.697 |  | 0.999 | 0.98 – 1.018 | 0.900 |
|  | CD158 | 0.999 | 0.988 – 1.011 | 0.915 |  | 1.001 | 0.988 – 1.013 | 0.924 |

**Supplementary table 5**. Association between NK cell phenotypes and time to culture conversion among TB/HIV co–infected participants (n=39)

|  |  | **Bivariable** | | |  | **Multivariable** | | |
| --- | --- | --- | --- | --- | --- | --- | --- | --- |
|  | **Cell marker** | **HR** | **CI** | **p–value** |  | **aHR** | **CI** | **p–value** |
| % Total NK cells |  | 0.982 | 0.958 – 1.005 | 0.128 |  | 0.993 | 0.968 – 1.020 | 0.621 |
|  |  |  |  |  |  |  |  |  |
| % CD56^bright^ |  | 0.951 | 0.882 – 1.026 | 0.196 |  | 0.858 | 0.768 – 0.959 | **0.007** |
| % CD56^dim^ |  | 0.99 | 0.969 – 1.012 | 0.372 |  | 0.987 | 0.962 – 1.013 | 0.326 |
| % CD56^dim^CD16^–^ |  | 1.001 | 0.981 – 1.022 | 0.891 |  | 1.006 | 0.982 – 1.031 | 0.622 |
| % CD56^neg^ |  | 1.024 | 0.996 – 1.052 | 0.089 |  | 1.023 | 0.997 – 1.05 | 0.080 |
|  |  |  |  |  |  |  |  |  |
| % CD56^bright^ | NKG2C | 0.991 | 0.974 – 1.008 | 0.277 |  | 0.996 | 0.980 – 1.013 | 0.672 |
|  | NKG2D | 1.009 | 0.993 – 1.024 | 0.272 |  | 1.009 | 0.994 – 1.024 | 0.251 |
|  | NKp30 | 1.011 | 0.993 – 1.029 | 0.25 |  | 1.007 | 0.987 – 1.026 | 0.505 |
|  | NKp44 | 1.014 | 0.970 – 1.06 | 0.532 |  | 1.002 | 0.956 – 1.051 | 0.932 |
|  | NKB1 | 0.965 | 0.920 – 1.012 | 0.142 |  | 0.990 | 0.940 – 1.043 | 0.706 |
|  | NKG2A | 1.007 | 0.986 – 1.030 | 0.508 |  | 0.988 | 0.964 – 1.013 | 0.339 |
|  | NKp46 | 0.997 | 0.977 – 1.018 | 0.794 |  | 0.975 | 0.952 – 0.999 | **0.045** |
|  | CD158 | 0.977 | 0.938 – 1.019 | 0.282 |  | 1.007 | 0.961 – 1.056 | 0.760 |
|  |  |  |  |  |  |  |  |  |
| % CD56^dim^ | NKG2C | 1.006 | 0.992 – 1.021 | 0.411 |  | 1.013 | 0.997 – 1.03 | 0.119 |
|  | NKG2D | 0.952 | 0.911 – 0.994 | **0.026** |  | 0.953 | 0.905 – 1.003 | 0.066 |
|  | NKp30 | 0.997 | 0.977 – 1.017 | 0.751 |  | 0.993 | 0.972 – 1.014 | 0.485 |
|  | NKp44 | 0.965 | 0.4 – 2.325 | 0.936 |  | 0.682 | 0.282 – 1.65 | 0.396 |
|  | NKB1 | 0.993 | 0.976 – 1.01 | 0.394 |  | 0.998 | 0.981 – 1.015 | 0.811 |
|  | NKG2A | 0.993 | 0.972 – 1.015 | 0.540 |  | 0.983 | 0.96 – 1.005 | 0.133 |
|  | NKp46 | 0.997 | 0.969 – 1.026 | 0.851 |  | 0.981 | 0.951 – 1.011 | 0.213 |
|  | CD158 | 0.994 | 0.98 – 1.009 | 0.459 |  | 0.999 | 0.984 – 1.014 | 0.872 |

**Supplementary table 6.** Association between NK cell phenotypes and disease severity (lung cavitation) in the total cohort (n=55)

|  | **Cell marker** | **Bivariable** | | |  | **Multivariable** | | |
| --- | --- | --- | --- | --- | --- | --- | --- | --- |
|  |  | **OR** | **CI** | **p–value** |  | **aOR** | **95% CI** | **p–value** |
| % Total NK cells |  | 0.993 | 0.962 – 1.025 | 0.649 |  | 0.991 | 0.960 – 1.024 | 0.605 |
|  |  |  |  |  |  |  |  |  |
| % CD56^bright^ |  | 0.924 | 0.842 – 1.015 | 0.099 |  | 0.921 | 0.834 – 1.016 | 0.101 |
| %CD56^dim^ |  | 1.027 | 0.991 – 1.064 | 0.140 |  | 1.027 | 0.991 – 1.065 | 0.144 |
| %CD56^dim^CD16^–^ |  | 0.974 | 0.945 – 1.003 | 0.083 |  | 0.971 | 0.942 – 1.001 | 0.059 |
| % CD56^neg^ |  | 1.032 | 0.985 – 1.082 | 0.190 |  | 1.041 | 0.989 – 1.095 | 0.124 |
|  |  |  |  |  |  |  |  |  |
| % CD56^bright^ | NKG2C | 0.980 | 0.954 – 1.006 | 0.128 |  | 0.979 | 0.953 – 1.006 | 0.127 |
|  | NK2D | 1.011 | 0.988 – 1.034 | 0.348 |  | 1.014 | 0.989 – 1.040 | 0.274 |
|  | NKp30 | 1.028 | 0.998 – 1.058 | 0.068 |  | 1.036 | 1.002 – 1.071 | **0.039** |
|  | NKp44 | 1.039 | 0.956 – 1.128 | 0.371 |  | 1.050 | 0.957 – 1.152 | 0.302 |
|  | NKB1 | 1.053 | 0.940 – 1.178 | 0.372 |  | 1.052 | 0.938 – 1.179 | 0.388 |
|  | NKG2A | 1.015 | 0.981 – 1.051 | 0.387 |  | 1.018 | 0.981 – 1.057 | 0.343 |
|  | NKp46 | 0.993 | 0.961 – 1.026 | 0.672 |  | 0.992 | 0.958 – 1.028 | 0.673 |
|  | CD158 | 1.068 | 0.956 – 1.194 | 0.246 |  | 1.068 | 0.955 – 1.194 | 0.248 |
|  |  |  |  |  |  |  |  |  |
| % CD56^dim^ | NKG2C | 0.997 | 0.973 – 1.022 | 0.840 |  | 0.997 | 0.972 –1.023 | 0.824 |
|  | NKG2D | 1.015 | 0.965 – 1.067 | 0.557 |  | 1.020 | 0.966 – 1.077 | 0.470 |
|  | NKp30 | 1.005 | 0.976 – 1.034 | 0.760 |  | 1.005 | 0.974 – 1.034 | 0.755 |
|  | NKp44 | 0.552 | 0.174 – 1.745 | 0.311 |  | 0.558 | 0.158 – 1.971 | 0.365 |
|  | NKB1 | 1.041 | 0.987 – 1.098 | 0.135 |  | 1.040 | 0.988 – 1.094 | 0.132 |
|  | NKG2A | 0.983 | 0.952 – 1.016 | 0.318 |  | 0.982 | 0.950 – 1.015 | 0.276 |
|  | NKp46 | 0.971 | 0.937 – 1.006 | 0.103 |  | 0.958 | 0.921 – 0.997 | **0.035** |
|  | CD158 | 1.026 | 0.988 – 1.066 | 0.177 |  | 1.025 | 0.988 – 1.064 | 0.184 |

**Supplementary table 7**. Association between NK cell phenotypes and disease severity (lung cavitation) among TB/HIV co–infected participants (n=35)

|  | **Cell marker** | **Univariable** | | |  | **Multivariable** | | |
| --- | --- | --- | --- | --- | --- | --- | --- | --- |
|  |  | **OR** | **CI** | **p–value** |  | **aOR** | **CI** | **p–value** |
| % Total NK cells |  | 0.996 | 0.958 – 1.036 | 0.838 |  | 0.994 | 0.952 – 1.038 | 0.778 |
|  |  |  |  |  |  |  |  |  |
| % CD56^bright^ |  | 0.913 | 0.806 – 1.035 | 0.154 |  | 0.840 | 0.685 – 1.031 | 0.096 |
| %CD56^dim^ |  | 1.009 | 0.966 – 1.053 | 0.700 |  | 1.009 | 0.965 – 1.055 | 0.697 |
| %CD56^dim^CD16^–^ |  | 0.990 | 0.954 – 1.027 | 0.598 |  | 0.988 | 0.950 – 1.027 | 0.540 |
| % CD56^neg^ |  | 1.023 | 0.971 – 1.078 | 0.398 |  | 1.031 | 0.972 – 1.095 | 0.308 |
|  |  |  |  |  |  |  |  |  |
| % CD56^bright^ | NKG2C | 0.987 | 0.954 – 1.020 | 0.433 |  | 0.983 | 0.949 – 1.019 | 0.353 |
|  | NKG2D | 1.003 | 0.969 – 1.038 | 0.876 |  | 1.004 | 0.969 – 1.040 | 0.825 |
|  | NKp30 | 1.029 | 0.990 – 1.069 | 0.142 |  | 1.035 | 0.993 – 1.079 | 0.108 |
|  | NKp44 | 1.028 | 0.941 – 1.123 | 0.538 |  | 1.037 | 0.939 – 1.144 | 0.474 |
|  | NKB1 | 1.068 | 0.916 – 1.244 | 0.402 |  | 1.069 | 0.911 – 1.254 | 0.416 |
|  | NKG2A | 1.011 | 0.974 – 1.049 | 0.564 |  | 1.016 | 0.973 – 1.060 | 0.469 |
|  | NKp46 | 1.001 | 0.965 – 1.039 | 0.962 |  | 1.001 | 0.961 – 1.043 | 0.959 |
|  | CD158 | 1.099 | 0.938 – 1.287 | 0.244 |  | 1.109 | 0.939 –1.311 | 0.223 |
|  |  |  |  |  |  |  |  |  |
| % CD56^dim^ | NKG2C | 0.990 | 0.961 – 1.020 | 0.518 |  | 0.990 | 0.961 – 1.020 | 0.513 |
|  | NKG2D | 0.956 | 0.870 – 1.049 | 0.342 |  | 0.960 | 0.870 – 1.058 | 0.407 |
|  | NKp30 | 1.002 | 0.966 – 1.040 | 0.895 |  | 0.999 | 0.962 – 1.038 | 0.976 |
|  | NKp44 | 0.591 | 0.152 – 2.297 | 0.447 |  | 0.571 | 0.133 – 2.462 | 0.453 |
|  | NKB1 | 1.058 | 0.987 – 1.134 | 0.114 |  | 1.063 | 0.986 – 1.146 | 0.112 |
|  | NKG2A | 0.970 | 0.933 – 1.009 | 0.126 |  | 0.968 | 0.930 – 1.008 | 0.113 |
|  | NKp46 | 0.943 | 0.892 – 0.998 | **0.044** |  | 0.937 | 0.883 – 0.994 | **0.032** |
|  | CD158 | 1.046 | 0.990 – 1.105 | 0.108 |  | 1.050 | 0.991 – 1.113 | 0.100 |
